# Metabolite analysis of wheat dough fermentation incorporated with buckwheat

**DOI:** 10.1002/fsn3.1720

**Published:** 2020-06-23

**Authors:** Binchen Wang, Lin Xiao, Duo Chai, Yumeng Jiang, Meiting Wang, Xianbing Xu, Chongwei Li, Liang Dong

**Affiliations:** ^1^ School of Food Science and Technology Dalian Polytechnic University Dalian Liaoning China; ^2^ National Engineering Research Center of Seafood Dalian Liaoning China; ^3^ Engineering Research Center of Agricultural Microbiology Technology Ministry of Education Heilongjiang University Harbin Heilongjiang China

**Keywords:** buckwheat, dough fermentation, metabolite analysis, volatile compounds

## Abstract

Dough fermentation represents an important developmental stage in the manufacturing process. In this study, volatile and nonvolatile metabolite analysis were carried out to investigate time‐dependent metabolic changes in the course of wheat dough fermentation incorporated with buckwheat based on gas chromatography–mass spectrometry (GC/MS). A total of 70 nonvolatile metabolites were identified, covering a broad spectrum of polar (e.g., amino acids, sugars, sugar alcohols, and acids) and nonpolar (e.g., fatty acid methyl esters, free fatty acids, and sterols) low molecular weight dough constituents. Meanwhile, sixty‐four volatile metabolites comprising aldehydes, ketones, alcohols, organic acids, aromatic compounds, and furans were identified using solid‐phase micro‐extraction combined with GC–MS. Some differences may exist in the volatile composition between fermented and unfermented dough. Statistical assessment of the nonvolatile data via principal component analysis demonstrated that the metabolic changes during the mixed dough fermentation are reflected by time‐dependent shifts of polar nonvolatile metabolites. And some potential nutritional markers, such as amino acids and sugars, could be developed to optimize and control the industrial dough fermentation incorporated with buckwheat.

## Graphical abstract

This study analyzed both volatile and non‐volatile compounds of wheat dough fermentation incorporated with buckwheat.

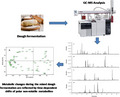

## 1 INTRODUCTION

Wheat bread is a popular food consumed worldwide because of its substantial intake of several nutrients. In recent years, with big concern about human health and relative healthy food, the poor nutritional quality of wheat bread catches much of attentions (Dewettinck et al., 2008; Moroni, Zannini, Sensidoni, & Arendt, 2012; Salehi, 2019). Additionally, based on its high content of essential nutrients, buckwheat has attracted increasing attention as alternative crop for the production of functional foods (Przygodzka, Zieliński, Ciesarová, Kukurová, & Lamparski, 2016; Raikos, Neacsu, Russell, & Duthie, 2014; Skrabanja et al., 2004). Meanwhile, it is also regarded as gluten‐free food, satifies the demands of people who are suffering from coeliac disease (De Francischi, Salgado, & Da Costa, 1994). Moreover, buckwheat contains some antioxidants, mainly rutin and quercetin, which was claimed to be effective to strengthen capillary blood vessels and reduce diabetes II (Li & Zhang, 2001; Watanabe, 1998). Nonetheless, the whole flour buckwheat bread products were found to be bad palatability, digestibility, and baking performances due to buckwheat's content of phytate and tannins, which may decrease the digestibility of buckwheat proteins and confer bitterness to buckwheat products (Li & Zhang, 2001). Therefore, incorporation of buckwheat is considered as a win‐win solution for enhancing the overall quality of wheat bread and the palatability of buckwheat bread.

Bread production consists of two main processes: dough fermentation and baking. Particularly, dough fermentation represents an important developmental stage in the manufacturing process (Birch, Petersen, Arneborg, & Hansen,^2013^ ; Birch, Petersen, & Hansen 2013; Hansen & Schieberle, 2005; Pico, Martínez, Bernal, & Gómez, 2017). This stage is characterized by numerous metabolic processes leading to distinct and time‐dependent alterations in metabolite levels. It is also found to be an effective way to enhance the palatability, digestibility and baking performances of buckwheat. Therefore, it is not surprising that metabolomics has proven to be a suitable tool for the investigation of this process. GC‐based metabolite profiling techniques have been applied to comparative investigations of many industrial production processes, such as malting, germination of rice (Frank, Meuleye, Miller, Shu, & Engel, 2007; Frank et al., 2007; Gorzolka, Lissel, Kessler, Loch‐Ahring, & Niehaus, 2012). Meanwhile, metabolite profiling is also considered to provide valuable data for fermentation‐driven metabolic engineering of nutritionally important metabolites in grain food. And metabolites profiling of sourdough fermented wheat flour and rye bread have been performed (Koistinen et al., 2018; Ripari, Cecchi, & Berardi, 2016).

Generally, the incorporation of buckwheat dough in wheat bread has the potential to improve the overall quality of the bread. However, no volatile and nonvolatile metabolomics‐based investigations of wheat dough fermentation incorporated with buckwheat have been conducted. The aim of this study was: from the perspective of food nutrition, to apply metabolic methods to wheat in the dough fermentation process incorporated with buckwheat enabling the analysis of a broad spectrum of low molecular weight metabolites; to identify and to quantify major contributors to the time‐dependent metabolic dynamic changes in the mixed dough fermentation; to compare volatile composition differences between fermented and unfermented mixed dough.

## 2 MATERIALS AND METHODS

### 2.1 Chemicals

Wheat and buckwheat flour was purchased from a local supermarket in Dalian, Liaoning, China. Standard chemicals: analytical grade (pentanal, hexanal, heptanal, octanal, nonanal, decanal, 1‐penten‐3‐ol, pentanol, hexanol, heptanol, octanol, toluene, ethyl benzene, p‐xylene, styrene, 2‐pentyl‐furan, and C4–C20 n‐alkanes) were purchased from Sigma‐Aldrich. Internal standards (tetracosane, p‐chloro‐L‐phenylalanine, 5R‐cholestan‐3β‐ol, Phenyl‐β‐D‐glucopyranoside) were purchased from Aladdin.

### 2.2 Preparation of dough

Basically, dough was prepared according to AACCI Approved Method 10‐10.03 with the following formula: 80.0 g of wheat flour, 20.0 g of buckwheat flour, 1.5% (w/w) sodium chloride, 1.0% (w/w) dry yeast and 52.0% (v/w) water. The gradients were mixed in a 100 g pin bowl mixer for 3 min 50 s. Then, dough was fermented for 2 hr (32°C; humidity of 75%). Samples were taken from 3 different part of dough every 20 min, then put them in liquid nitrogen and freeze‐dried for 48 hr. All dried samples were milled and stored at −30°C until analysis.

### 2.3 Volatile metabolite analysis

#### 2.3.1 Solid‐phase micro‐extraction sampling

To identify dough volatile compounds, the headspace (HS) SPME technique was employed. For this study, the temperature was maintained at 18°C ± 1°C to avoid formation of flavor compound artifacts. The mixed dough slurry was prepared by blending flour prepared before (3.0 g), 0.5 mol/L sodium chloride solution (2 ml), in a 20 ml flask with a cap and Teflon‐faced silicone rubber septa (Supelco, Co.). The flask containing the sodium chloride solution and flour was placed on a magnetic stirring plate (model PC‐220) and stirred at 1,100 r min^−1^ for 20 min. A SPME fiber (DVB/CAR/PDMS) was then exposed to the headspace of the dough slurry for 1 hr in a water bath at 18°C ± 1°C (Dong et al., 2013).

#### 2.3.2 GC–MS analysis

Agilent 7890A GC‐5975C MSD was used to identify the volatile compounds of the mixed dough. The oven temperature program was set at 35^○^C for 3 min and then to 280^○^C at 5^○^C/min, with a cycle time of 52 min. The temperatures of the injector and ion source were 250^○^C and 230^○^C, respectively. Electron ionization mass spectra were recorded at 70 eV. The mass spectrometer was operated in full scan mode, with an *m*/*z* range from 40 to 400 amu and a scan time of 0.25 s. Helium was used as a carrier gas at a flow rate of 1 ml/min. The HP‐5MS column had the following dimensions: length: 30 m; internal diameter: 0.25 mm; and film thickness: 0.25 μm. Retention indices (RI) were calculated for each compound using homologous series of C4–C20 n‐alkanes (Dong et al., 2015).

### 2.4 Nonvolatile metabolite analysis

Extraction and fractionation of freeze‐dried mixed dough flour were performed in accordance with the procedure developed a previously described procedure developed by Frank et al. (2007), with little minor modifications. The substances were divided into two fractions, namely, polar fractions (amino acids and sugars) and nonpolar fractions (fatty acid methyl ester [FAME], sterols and free fatty acid [FFA]). Samples (300 mg) were added to 5 ml of 80% (v/v) methanol solution for 15 min to extract the amino acids. The mixture was centrifuged for 10 min at 10,000 rpm. Then, 1 ml of the supernatant was poured into a glass vial, and 10 μl of p‐chloro‐L‐phenylalanine in deionized water (0.3 mg/ml) was added as a quantification standard. The methanol solution in the upper layer was dried with nitrogen and used for the subsequent steps. The remaining solid was dissolved into 360 μl of acetonitrile, then 40 μl of MTBSTFA (N‐tert‐Butyldimethylsilyl‐N‐methyltrifluoroacetamide) was added and heated at 70°C for 30 min for the silylation of amino acids. As for the sugars, 100 mg of the samples were extracted using the method discussed above, but the supernatant was taken at the volume of 50 μl. Subsequently, 30 μl of phenyl β‐D‐glucopyranoside (1.6 mg/ml in water) was added to the extract as a quantification standard and then dried with nitrogen. The dry residue was dissolved again in 250 μl of acetonitrile, and 50 μl of TSIM (N‐[Trimethylsilyl]imidazole) was added for the silylation of sugars at 70°C for 30 min.

**FIGURE 1:**
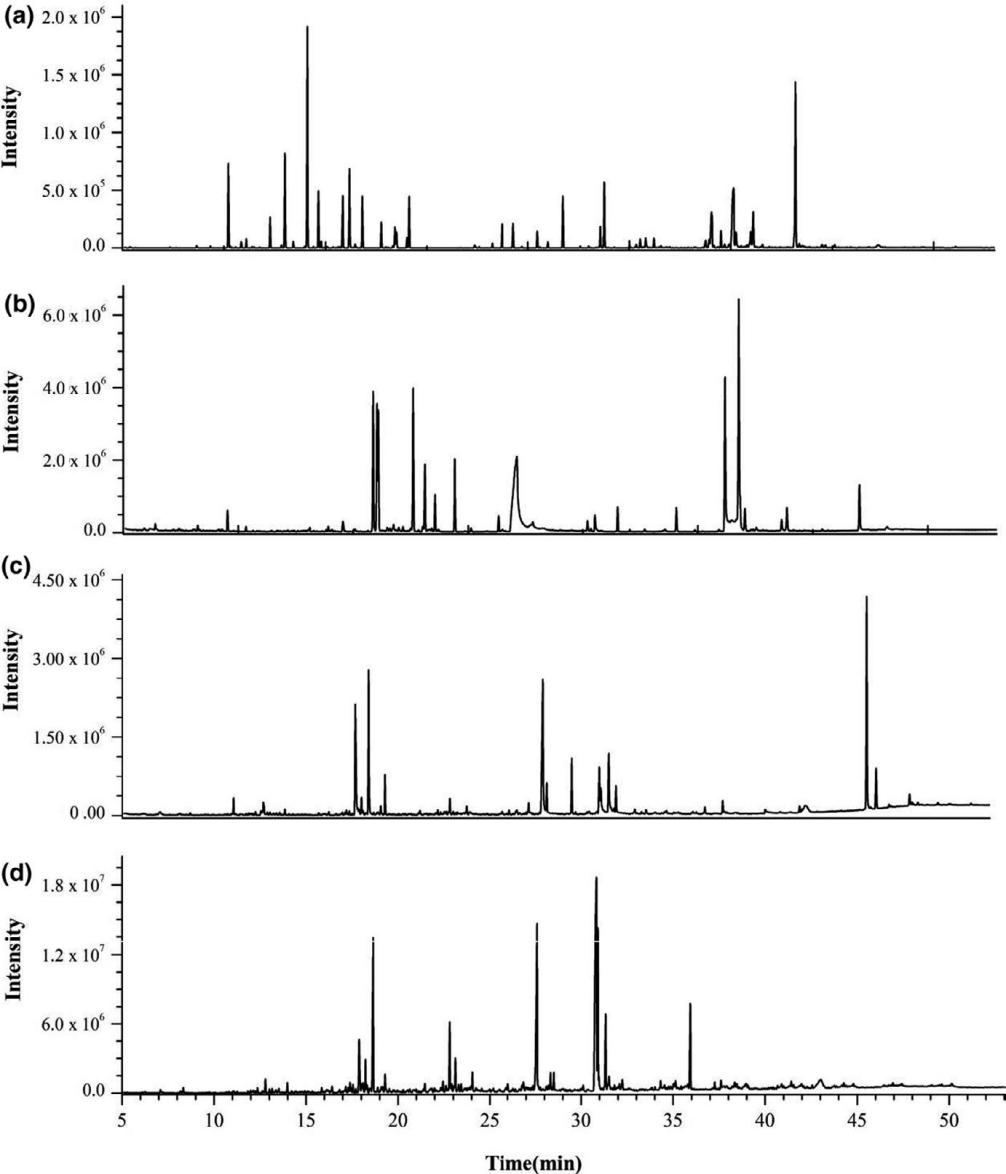
Total ion current chromatograms based on GC/MS of different fractions in unfermented mixed flour: (a) amino acid; (b) sugar; (c) free fatty acid; (d) fatty acid methyl ester

The extraction of the nonpolar fraction was performed by following completely the method of Frank (Frank et al., 2007). The 100 mg sample was extracted with dichloromethane for 15 min, and then the transesterified FAME, sterols, and FFA were separated by transesterification and solid‐phase extraction. Finally, silylation was carried out. All of the obtained fractions were analyzed by gas chromatography–mass spectrometry (GC/MS). The GC/MS conditions were in agreement with previously described procedures. And the total ion current chromatograms of different fractions were shown in Figure 1.

### 2.5 Identification of compounds

Dough constituents both volatile and nonvolatile were identified by comparing retention times and mass spectra with those of reference compounds, or by comparing mass spectra with the entries of the mass spectra library NIST2.2.

### 2.6 Statistical analysis

Dough samples at different fermentation stages were analyzed in triplicate. Relative content of each volatile compound was standardized. Principal component analysis (PCA) was performed using XLSTAT 7.5.2 (Addinsoft).

## 3 RESULTS AND DISCUSSION

### 3.1 Nonvolatile metabolite analysis in dough fermentation process incorporated with buckwheat

The applied GC metabolite profiling approach allowed the detection of six fractions during the whole dough fermentation process incorporated with buckwheat, including amino acids, sugar and sugar alcohols, acids, fatty acid methyl esters, free fatty acids, and sterols. In total, 70 peaks were identified on the basis of retention times and mass spectrometric data from authentic reference compounds and/or from MS libraries, which was divided into two parts: polar (Table 1) and nonpolar fractions (Table 2).

**TABLE 1:** Total nonvolatile polar metabolites identified during dough fermentation incorporated with buckwheat

No.	Compound	No.	Compound	No.	Compound
Amino acids		17	Tyrosine	33	melibiose
1	Alanine	18	Tryptophan	Acids	
2	Glycine	19	Cysteine	46	Lactic acid
3	Proline	Sugars and sugar alcohols		47	Hexanoic acid
4	Leucine	20	Xylitol	48	Glycolic acid
5	Isoleucine	21	Fructofuranose	49	oxalic acid
6	Proline	22	D‐mannose	50	3‐hydroxybutyric acid
7	Methionine	23	D‐furanose	51	Octanoic acid
8	Serine	24	L‐sorbose	52	Nonanoic acid
9	Threonine	25	D‐glucose		
10	Phenylalanine	26	D‐glucitol		
11	Aspartic acid	27	Inositol		
12	Glutamate	28	Glyceryl glycoside		
13	Asparagine	29	sucrose		
14	Lysine	30	maltose		
15	Glutamine	31	D‐(+)‐Cellobiose		
16	Histidine	32	Trehalose		

**TABLE 2:** Total nonvolatile nonpolar metabolites identified during dough fermentation incorporated with buckwheat

No.	Compound	No.	Compound	No.	Compound
FAME		Free fatty acids		Sterols	
34	C15:0	53	C12:0	65	cholesterol
35	C16:1	54	C14:0	66	ergosterol
36	C16:0	55	C15:0	67	campesterol
37	C17:0	56	C16:1	68	β‐sitosterol
38	C18:2	57	C16:0	69	stigmastanol
39	C18:1	58	C17:0	70	fucosterol
40	C18:0	59	C18:2		
41	C20:1	60	C18:1		
42	C20:0	61	C18:1(1)		
43	C22:0	62	C18:0		
44	C24:0	63	C20:1		
45	C26:0	64	C20:0		

**FIGURE 2:**
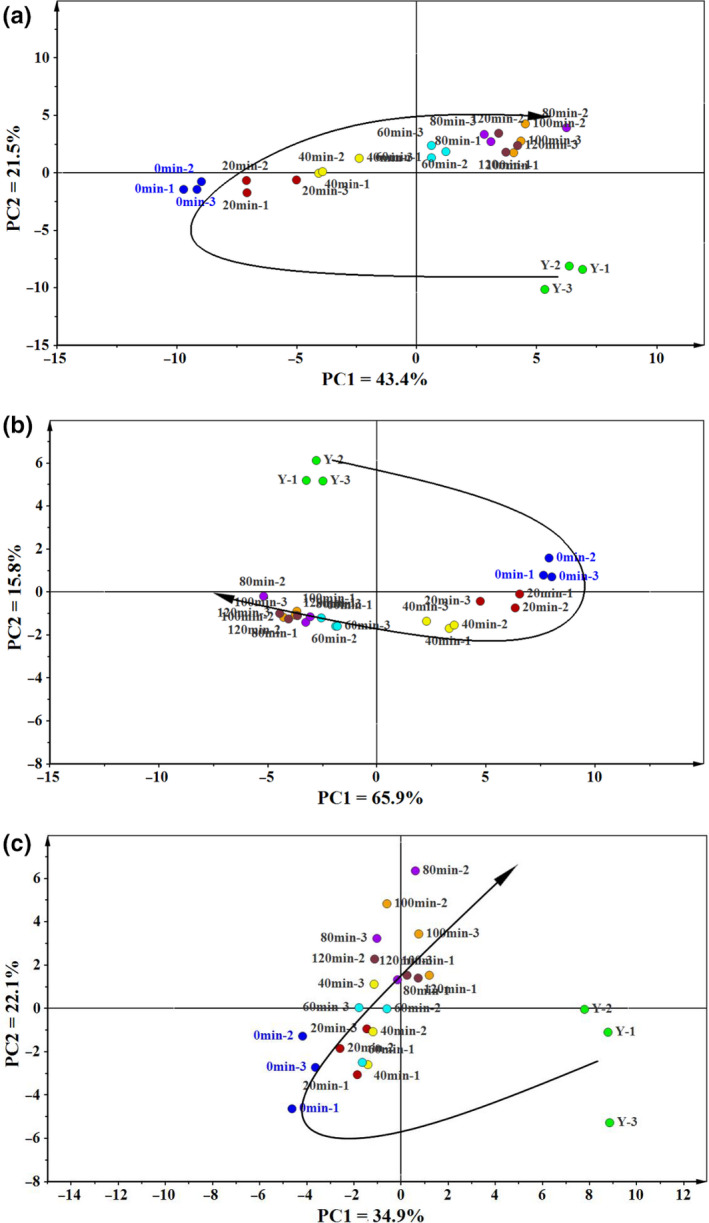
Score scatter plot of nonvolatile metabolites from dough fermentation incorporated with buckwheat to the PC1 and PC2. (a) combined fractions score; (b) polar fraction score; (c) nonpolar fraction score

Multivariate analysis was conducted by means of principal component analysis (PCA) for polar, nonpolar, and combined fractions. The scores plots obtained from the data on mixed dough at different stages of the fermentation process are shown in the Figure 2a–c. For the combined fractions, the first two principal components PC1 and PC2 explained 65% of the total variation seen in the Figure 2a. Compared to the nonpolar fractions, the scores plots of the polar fraction containing the metabolite profiles on amino acids, sugars, sugar alcohols, and acids exhibited an even more pronounced variance in the course of mixed dough fermentation (Figure 2b). For the nonpolar fraction, a clear separation was not observed, which gather in the center part of the scores plot (Figure 2c). Both the nonpolar and the polar fractions showed U‐shape patterns of the scores plots during the fermentation progress.

**FIGURE 3:**
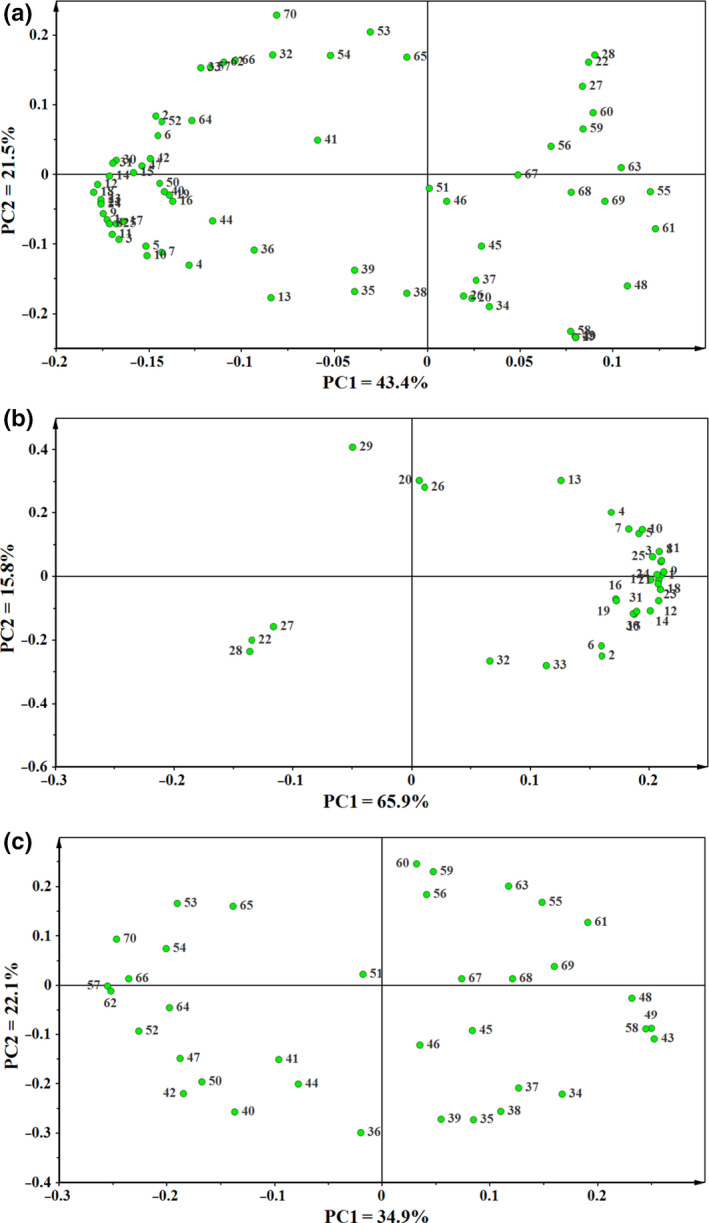
Loading scatter plot of nonvolatile metabolites from dough fermentation incorporated with buckwheat to the PC1 and PC2. (a) combined fractions score; (b) polar fraction score; (c) nonpolar fraction score

Analysis of the PCA loadings taking into account the data of 70 identified peaks detected in all the fractions resulted in the loadings plot shown in Figure 3a. For the combined fractions, the polar compounds were found to be major contributors to the separation along the first principal component, whereas predominantly the nonpolar metabolites were responsible for the separation along the second PC. This result reflects the more pronounced influence of the polar constituents on the separation of dough during the fermentation process. In order to assess the major drivers for the time‐dependent separation of the fermented dough samples, the PCA loading scores for each single fraction were analyzed (Figure 3b–c). The results show the polar compounds were also found to be major contributors to the time‐dependent separation of the fermented mixed dough samples. The later stages of dough fermentation (60–120 min) showed obvious separation from the former ones, with d‐mannose, inositol, glyceryl glycoside being the most variable compounds (Figure 3b). As to nonpolar compounds, less contributions were made to the time‐dependent separation. They are evenly distributed in the loading plot (Figure 3c).

### 3.2 Changes of polar nonvolatile metabolite during dough fermentation incorporated with buckwheat

**FIGURE 4:**
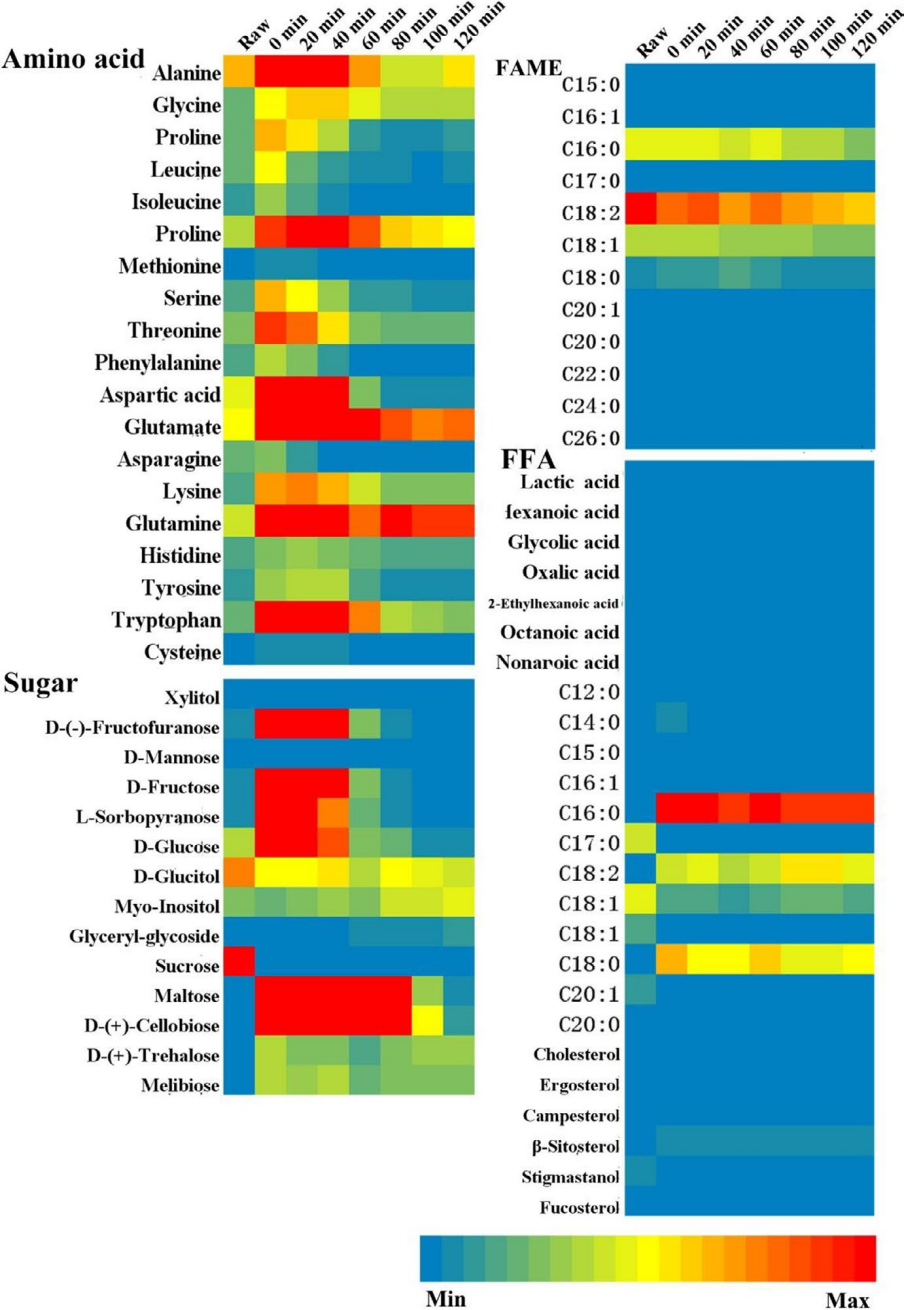
Heatmap of nonvolatile metabolites from dough fermentation incorporated with buckwheat

In order to more intuitively see the variation tendency of metabolites at different mixed dough fermentation stage, a heat map was presented for the nonpolar and polar dough metabolite profiles (Figure 4). As it was shown in Figure 4, the polar compounds exhibited much more pronounced alterations in the course of dough fermentation, which is expressed by the significant color changes in the heatmap on the polar dough metabolite profile.

For amino acids fractions, significantly increased levels of most of identified amino acids were observed at the beginning of the fermentation (0–40 min) due to proteolytical enzyme activities of the grain, except for methionine and cysteine (Azizi, Azizi, Moogouei, & Rajaei, 2020; Nkhata, Ayua, Kamau, & Shingiro, 2018). And the highest levels of all amino acids also appeared at this stage. With the fermentation process went on, yeast began to use amino acids to rapidly multiply, causing a rapid drop in all the amino acid content after 40 min.

Generally, similar changes were observed for sugar fractions at the beginning step of mixed dough fermentation. Most of sugars identified exhibited an increased level from 0 to 40 min of the fermentation process, the significantly increased levels of mono‐ and disaccharides, for example, glucose, fructose, sorbose, cellobiose, and maltose were observed (Figure 4). The reasons for these increased phenomenons were also considered as the contribution of proteolytical enzyme activities of the grain (Barber, Prieto, & Collar, 1989). After 40 min of fermentation, similar to the expected changes observed for sugars, the unitization of yeast reproductions leads to the observed decrease in mono‐saccharides, such as glucose and fructose. Meanwhile, the time for decreased levels of disaccharides like cellobiose and maltose was later than mono‐saccharides due to the diauxie of yeast cell.

The levels of acids identified were found to be low and constant during the whole fermentation process. But they were very crucial to the dough flavor because of the esterification with alcohols to form esters, which were major contributor to the dough flavor (Pico, Bernal, & Gómez, 2015).

Generally, the polar amino acids and sugars exhibited the potential to serve as nutritional markers for the wheat dough fermentation incorporated with buckwheat, based on their remarkable change in the fermentation process. Their potential application would meet the bakers’ demand to maintain the maximum nutritional level both in quality and quantity.

### 3.3 Changes of nonvolatile nonpolar metabolite during dough fermentation incorporated with buckwheat

Compared to the relative big changes in polar contents, the nonpolar compounds exhibited little alterations in the course of fermentation. As it was shown in Figure 4, the levels of major fatty acid methyl esters (FAME) including saturated C16, saturated C18, and all the unsaturated C18 were shown decreased during the whole fermentation process. Other FAMEs showed lower levels and no change at same stage of fermentation. The reason for this phenomenon was that both of saturated and unsaturated C16–C18 fatty acid was the major composition of triglycerides both in wheat and buckwheat, and an enzymatic degradation of triglycerides was also a contributor leading to the increasing levels of free fatty acids related, such as stearic acid, oleic acid, linoleic acid, and free hexadecyl fatty acid.

As mentioned above, the levels of free fatty acids increased slightly during the fermentation process, and the levels of other free fatty acids were low and constant at the same fermentation stage. Regarding the dynamic changes of sterols, dough exhibited lower or constant contents or of the major sterols.

### 3.4 Changes of volatile metabolite during dough fermentation incorporated with buckwheat

Of the 64 volatile metabolites including 11 aldehydes, 15 alcohols, 8 ketones, 4 furans, 10 aromatic compounds, 16 acids, and esters were finally identified from the mixed dough during the whole fermentation process (Table 3). As it was shown in Table 3, all the aldehydes kept a low and relative constant level during fermentation. They were responsible for the fresh and slightly green notes of dough at a low concentration, and for the butter flavor at higher one (Nor Qhairul Izzreen, Petersen, & Hansen, 2016). Meanwhile, most of volatile alcohols had an increased level at the end of the fermentation compared with the beginning. And some of them exhibited a significantly increased level, such as ethanol, 2‐methyl‐1‐propanol, 3‐methyl‐1‐butanol, and 2‐methyl‐1‐butanol, because they were the by‐product of yeast cell reproduction. These volatile alcohols usually contribute a sweet and alcohol odor to the dough (Pico et al., 2017).

**TABLE 3:** Total volatile metabolites identified during dough fermentation incorporated with buckwheat

No.	Compound	RI	Identification	Peak area at different time(×10^5^)		
				0 min		120 min
Aldehydes						
1	Acetaldehyde	404	RI,MS,STD	5.92 ± 0.10		10.17 ± 0.61
2	3‐methyl‐Butanal	652	RI,MS,STD	4.32 ± 0.81		1.90 ± 0.43
3	2‐methyl‐Butanal	662	RI,MS,STD	3.12 ± 0.22		2.50 ± 0.42
4	Pentanal	699	RI,MS,STD	4.91 ± 0.63		ND
5	Hexanal	800	RI,MS,STD	72.00 ± 2.15		38.01 ± 10.75
6	Heptanal	901	RI,MS,STD	4.94 ± 0.46		4.61 ± 1.47
7	(Z)‐2‐Heptenal	958	RI,MS,STD	2.95 ± 0.43		3.93 ± 0.73
8	(E)‐2‐Octenal	1,060	RI,MS,STD	5.98 ± 0.95		7.22 ± 1.70
9	Nonanal	1,104	RI,MS,STD	8.52 ± 2.32		4.50 ± 1.52
10	Decanal	1,206	RI,MS,STD	2.16 ± 0.43		1.56 ± 0.29
11	(E,E)‐2,4‐Decadienal	1,317	RI,MS,STD	2.53 ± 0.44		2.24 ± 0.70
Alcohol						
12	Ethanol	427	RI,MS,STD	163.83 ± 2.16	394.18 ± 5.97	
13	1‐Propanol	555	RI,MS,STD	4.13 ± 0	14.16 ± 1.12	
14	2‐methyl‐1‐Propanol	625	RI,MS,STD	19.98 ± 2.04	112.08 ± 4.38	
15	1‐Penten‐3‐ol	684	RI,MS,STD	6.90 ± 0.26	1.94 ± 0.45	
16	3‐methyl‐1‐Butanol	736	RI,MS,STD	139.38 ± 18.10	477.61 ± 20.03	
17	2‐methyl‐1‐Butanol	739	RI,MS,STD	72.19 ± 11.09	188.64 ± 3.36	
18	1‐Pentanol	765	RI,MS,STD	63.16 ± 6.07	11.88 ± 2.52	
19	2,3‐Butanediol	788	RI,MS,STD	ND	5.22 ± 1.03	
20	1‐Hexanol	868	RI,MS,STD	148.98 ± 5.39	139.42 ± 12.95	
21	1‐Heptanol	970	RI,MS,STD	3.26 ± 0.19	8.18 ± 1.85	
22	1‐Octen‐3‐ol	980	RI,MS,STD	8.63 ± 0.71	9.15 ± 1.98	
23	(Z)‐2‐Octen‐1‐ol	1,067	RI,MS,STD	0.74 ± 0.16	0.71 ± 0.20	
24	1‐Octanol	1,071	RI,MS,STD	3.66 ± 0.26	4.40 ± 0.73	
25	(Z)‐3‐Nonen‐1‐ol	1,156	RI,MS,STD	4.98 ± 0.40	7.62 ± 2.25	
26	1‐Nonanol	1,173	RI,MS,STD	2.51 ± 0.52	3.83 ± 0.70	
Ketone						
27	2,3‐Butanedione	595	RI,MS,STD	1.87 ± 0.19	20.56 ± 0.04	
28	2‐Pentanone	685	RI,MS,STD	0.63 ± 0.19	0.61 ± 0.11	
29	2,3‐Pentanedione	698	RI,MS,STD	ND	2.59 ± 0	
30	Acetoin	713	RI,MS	1.31 ± 0.75	10.24 ± 0.36	
31	2‐Heptanone	891	RI,MS,STD	ND	4.25 ± 0.40	
32	2‐methyl‐3‐Octanone	985	RI,MS	2.20 ± 0.27	3.15 ± 0.16	
33	6‐methyl‐5‐Hepten‐2‐one	986	RI,MS,STD	7.01 ± 0.20	7.30 ± 0.56	
34	3‐Octen‐2‐one	1,040	RI,MS,STD	1.46 ± 0.29	0.85 ± 0.22	
Acid & Easter						
35	Acetic acid		RI,MS,STD	ND	3.68 ± 0.27	
36	Ethyl Acetate	612	RI,MS,STD	16.52 ± 3.25	73.38 ± 5.87	
37	Propanoic acid ethyl ester	709	RI,MS	ND	2.08 ± 0.07	
38	n‐Propyl acetate	708	RI,MS	ND	0.86 ± 0.05	
39	Isobutyl acetate	771	RI,MS	ND	1.92 ± 0.14	
40	Butanoic acid ethyl ester	802	RI,MS,STD	ND	4.63 ± 0.26	
41	2‐methyl‐Butanoic acid	861	RI,MS	ND	1.51 ± 0.20	
42	3‐methyl‐1‐Butanol acetate	876	RI,MS,STD	ND	5.61 ± 0.66	
43	2‐methyl‐1‐Butanol acetate	880	RI,MS,STD	ND	1.75 ± 0.08	
44	Butyrolactone	915	RI,MS	0.92 ± 0.25	0.53 ± 0.14	
45	Hexanoic acid ethyl ester	1,000	RI,MS,STD	2.07 ± 0.43	11.20 ± 1.10	
46	Acetic acid hexyl ester	1,011	RI,MS,STD	0.22 ± 0.05	0.57 ± 0.04	
47	Heptanoic acid ethyl ester	1,097	RI,MS,STD	ND	1.13 ± 0.13	
48	Octanoic acid ethyl ester	1,196	RI,MS,STD	ND	7.91 ± 0.33	
49	Nonanoic acid ethyl ester	1,296	RI,MS,STD	ND	0.79 ± 0.15	
50	Decanoic acid ethyl ester	1,396	RI,MS,STD	ND	0.63 ± 0.12	
Aromatic compound						
51	Ethylbenzene	855	RI,MS,STD	3.08 ± 1.16	2.70 ± 0.35	
52	p‐Xylene	865	RI,MS,STD	2.78 ± 1.10	1.52 ± 0.27	
53	Styrene	893	RI,MS,STD	1.75 ± 0.54	0.94 ± 0.08	
54	o‐Xylene	887	RI,MS,STD	4.91 ± 0.92		
55	Benzaldehyde	962	RI,MS,STD	5.32 ± 1.33	8.95 ± 0.77	
56	Benzyl alcohol	1,036	RI,MS,STD	0.34 ± 0.05	0.96 ± 0.09	
57	Benzeneacetaldehyde	1,045	RI,MS,STD	0.28 ± 0.03	1.63 ± 0.44	
58	Acetophenone	1,065	RI,MS,STD	0.54 ± 0.05	0.50 ± 0.07	
59	Phenylethyl Alcohol	1,116	RI,MS,STD	3.64 ± 0.60	41.22 ± 4.03	
60	4‐(1‐methylethyl)‐Benzaldehyde	1,239	RI,MS	0.80 ± 0.15	0.26 ± 0.09	
Furan						
61	dihydro‐5‐methyl‐2(3H)‐Furanone	958	RI,MS	0.40 ± 0.05	0.17 ± 0.26	
62	2‐pentyl‐Furan	993	RI,MS,STD	2.75 ± 0.58	2.92 ± 0.27	
63	5‐ethyldihydro‐2(3H)‐Furanone	1,057	RI,MS	1.21 ± 0.09	0.91 ± 0.20	
64	dihydro‐5‐pentyl‐2(3H)‐Furanone	1,363	RI,MS	0.83 ± 0.20	2.13 ± 0.50	

Abbreviations: MS, Identification by MS spectra; nd, Compound not detected in the sample; RI, Kovat's retention indexes, STD, Standard compound.

In this study, eight volatile ketones were identified. 2,3‐pentanedione and 2‐heptanone were only detected at the end of the fermentation. Moreover, similar changing pattern of volatile ketones was observed as aldehydes. All the volatile ketones kept a low and relative constant level during fermentation, except 2,3‐butanedione which showed an increased level at the end of the fermentation. This compound contributed a buttery, sulfurous, and pungent odor, which might be involved in the final flavor of the dough (Gassenmeier & Schieberle, 1995).

Acids and esters were most varied in types in dough fermentation. Ethyl acetate, butyrolactone, hexanoic acid ethyl ester, and acetic acid hexyl ester could be detected during the whole fermentation process. Other acids and esters could only be detected at the end of the fermentation, which contributed the major flavor differences between fermented dough and nonfermented one. Esters usually had a little fruit flavor at lower concentration, even which could be smelled at the end of the fermentation and also be formed by the esterification with alcohols during the fermentation as mentioned above (Birch, Petersen, Arneborg, et al., 2013; Birch et al., 2013; Gassenmeier & Schieberle, 1995). Levels of furan and aromatic compounds detected both at the beginning and the end of the fermentation were relative small. And there were no obvious differences between them. Furans usually have a sweet flavor, and aromatic compounds contribute a flower flavor at lower concentration.

## 4 CONCLUSION

Polar nonvolatile metabolites were the major contributors to the mixed dough fermentation time‐driven changes in the metabolic analysis. In addition to the representatives of flavor composition, nutritionally relevant metabolites are covered. They range from the nonpolar free fatty acids to the polar amino acids and sugars. And some potential nutritional markers, such as amino acids and sugars, could be developed in the future, which might finally be correlated to the contents of metabolites responsible for technological properties and the nutritional quality of mixed dough and be applied to optimize and control the dough fermentation. The polar amino acids and sugars exhibited the potential to serve as nutritional markers for the wheat dough fermentation incorporated with buckwheat. Of course, further study should be carried out to quantify each metabolite identified.

## CONFLICT OF INTEREST

Authors declare that they do not have any conflict of interest.

## ETHICAL STATEMENT

This study does not involve any human or animal testing.
